# Intraventricular thrombus and severe mitral regurgitation in the acute phase of takotsubo cardiomyopathy: two case reports

**DOI:** 10.1186/s13256-019-2081-0

**Published:** 2019-05-19

**Authors:** Daishi Nonaka, Hiroyuki Takase, Masashi Machii, Kazuto Ohno

**Affiliations:** Department of Internal Medicine, Enshu Hospital, JA Shizuoka Kohseiren, 1-1-1 Chuo, Naka-ku, Hamamatsu, Shizuoka 430-0929 Japan

**Keywords:** Takotsubo cardiomyopathy, Intraventricular thrombus, Mitral regurgitation

## Abstract

**Background:**

Takotsubo cardiomyopathy is characterized by chest symptoms, electrocardiographic changes, and new regional wall motion abnormality in the apical segment of the left ventricle in the absence of obstructive coronary artery disease. Particularly, apical ballooning is broadly recognized as the classic form of takotsubo cardiomyopathy. Although the prognosis of most patients with takotsubo cardiomyopathy is generally favorable, complications associated with the morphological features of transient apical ballooning are not uncommon.

**Case presentation:**

We describe two cases of transient complications in postmenopausal patients with takotsubo cardiomyopathy. Intraventricular thrombus was observed in Asian patient 1, and severe mitral regurgitation was observed in Asian patient 2. These complications were confirmed by transthoracic echocardiography immediately after typical takotsubo cardiomyopathy with apical ballooning was diagnosed. Anticoagulant therapy with heparin and warfarin was continued for 1 week in patient 1. After the therapy, complete resolution of the apical thrombus and recovery of systolic function of the left ventricle was observed by follow-up transthoracic echocardiography. In patient 2, transthoracic echocardiography indicated significant mitral regurgitation, which was caused by left ventricular tethering of the anterior mitral leaflet rather than left ventricular outflow tract obstruction or systolic anterior motion. Because the hemodynamic stability in patient 2 had been preserved, she was managed with conservative treatment. After approximately 1 month, follow-up transthoracic echocardiography revealed that mitral regurgitation had almost disappeared with complete resolution of left ventricular wall motion abnormalities.

**Conclusions:**

The presented cases indicated that important complications, such as intraventricular thrombus and severe mitral regurgitation, are associated with takotsubo cardiomyopathy in the acute phase. Because these complications are risk factors for developing a thromboembolic event or heart failure and/or pulmonary edema, timely and accurate identification of these complications is critical to achieving optimal clinical outcomes in patients with takotsubo cardiomyopathy.

## Background

Takotsubo cardiomyopathy (TCM), also known as stress cardiomyopathy or broken heart syndrome, is typically characterized by the morphological features of transient apical ballooning of the left ventricle. It occurs more often in postmenopausal women and is frequently associated with emotional, psychological, or physical stress preceding the presentation [1]. Commonly, patients have chest discomfort and electrocardiographic changes, such as ST-segment elevation or T-wave inversion mimicking acute myocardial infarction. Transthoracic echocardiography (TTE) or left ventriculography demonstrates hypokinesis or akinesis in the apical to middle segments of the left ventricle beyond a single vascular territory. In fact, coronary angiography shows normal coronary arteries independent of plaque rupture or myocardial ischemia with significant coronary obstruction [2]. Although left ventricular (LV) dysfunction is reversible and the long-term prognosis is generally excellent, several complications related to abnormal LV contraction may occur.

Recently, TCM has been recognized worldwide, and reports on patients with TCM have been increasing, but there are only a few reports on thrombosis and valvular disease complicated with TCM. We report two cases of patients with TCM with transient complications, such as formation of an intraventricular thrombus and severe mitral valve regurgitation (MR).

## Case presentation

### Patient 1

**Fig. 1:**
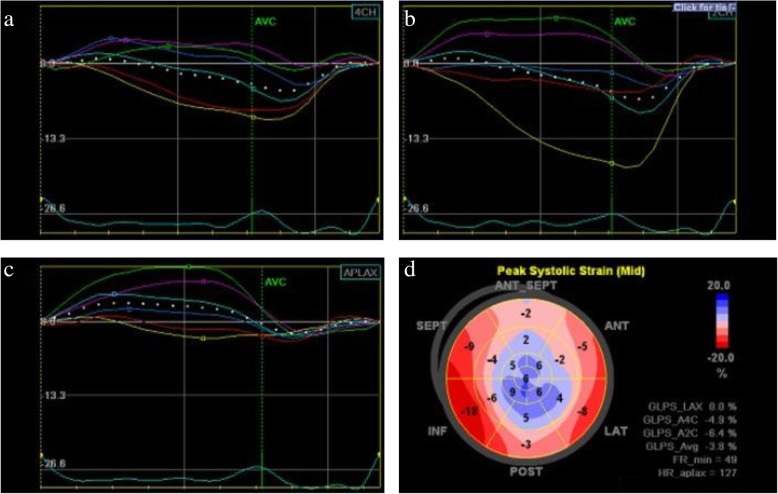
Longitudinal strain curves from four-chamber (**a**), two-chamber (**b**), and apical long-axis (**c**) views by two-dimensional transthoracic echocardiography on admission are used to generate bull’s-eye summary of the entire left ventricle (**d**). The bull’s-eye plot shows more impaired longitudinal strain in the apical segments

An 83-year-old Asian woman was admitted to our hospital with a chief complaint of pain in the left arm after a fall and was hospitalized with a diagnosis of left humeral fracture. The patient had a medical history of anxiety neurosis and reflux esophagitis, and she had been taking medications including etizolam (0.5 mg) and lansoprazole (15 mg). She had no smoking habit or alcohol consumption. She also had no family history or employment history of note. On examination at the time of admission, her height and weight were 1.45 m and 43.0 kg, respectively (body mass index, 20.5 kg/m^2^). Her blood pressure was 178/86 mmHg, pulse 99 beats/minute (regular), and respiratory rate of 18/minute with oxygen saturation of 96% on room air. Her body temperature was 37.2 °C. Her physiological examination revealed no abnormalities, with the exception of arm pain. She had alert consciousness, and her neurological examination result was normal. On the day following admission, she experienced sudden onset of chest pain and palpitations associated with cold sweats and shivering. On physical examination, her heart rate, blood pressure level, and oxygen saturation were 118 beats/minute, 119/75 mmHg, and 97%, respectively. She was afebrile, and neither heart murmurs nor abnormal breath sounds were heard. Her abdominal examination showed no notable findings. Edema was not detected in either lower limb. Her consciousness level was clear, and no apparent neurological deficit was observed. An electrocardiogram (ECG) showed ST-segment elevations in II, III, aVF, and V3–V6 leads. Her creatine kinase (CK) and N-terminal pro-B-type natriuretic peptide (NT-proBNP) levels were 519 U/L (normal range, 30–170 U/L) and 5435 pg/ml (< 125 pg/ml), respectively. Her qualitative troponin T was positive. Two-dimensional TTE using speckle tracking (Vivid E9; GE Healthcare, Horten, Norway) with an automated function imaging technique provided with the commercial imaging analysis software (EchoPAC; GE Healthcare) showed akinesis of the middle and apical segments of the left ventricle with typical apical ballooning and hyperkinesis of the basal segments (Fig. 1), whereas no thrombus was observed in the apex of the left ventricle. Emergent cardiac catheterization was performed, and apical akinesis of the left ventricle without explanatory severe coronary stenosis was observed during the procedure. On the basis of these findings, the patient was diagnosed with TCM.

**Fig. 2:**
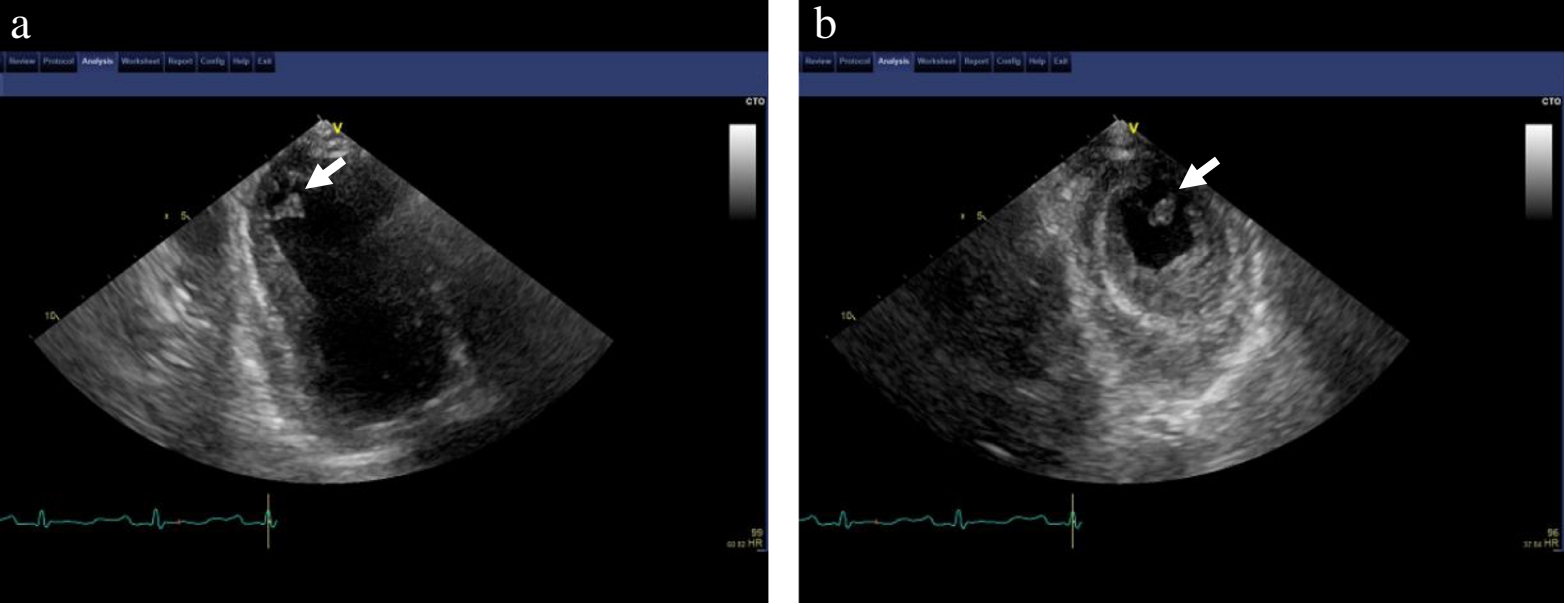
Transthoracic echocardiographic images. Long-axis (**a**) and short-axis (**b**) views on the seventh hospitalization day showing a thrombus in the apex of the left ventricle (*arrow*)

**Fig. 3:**
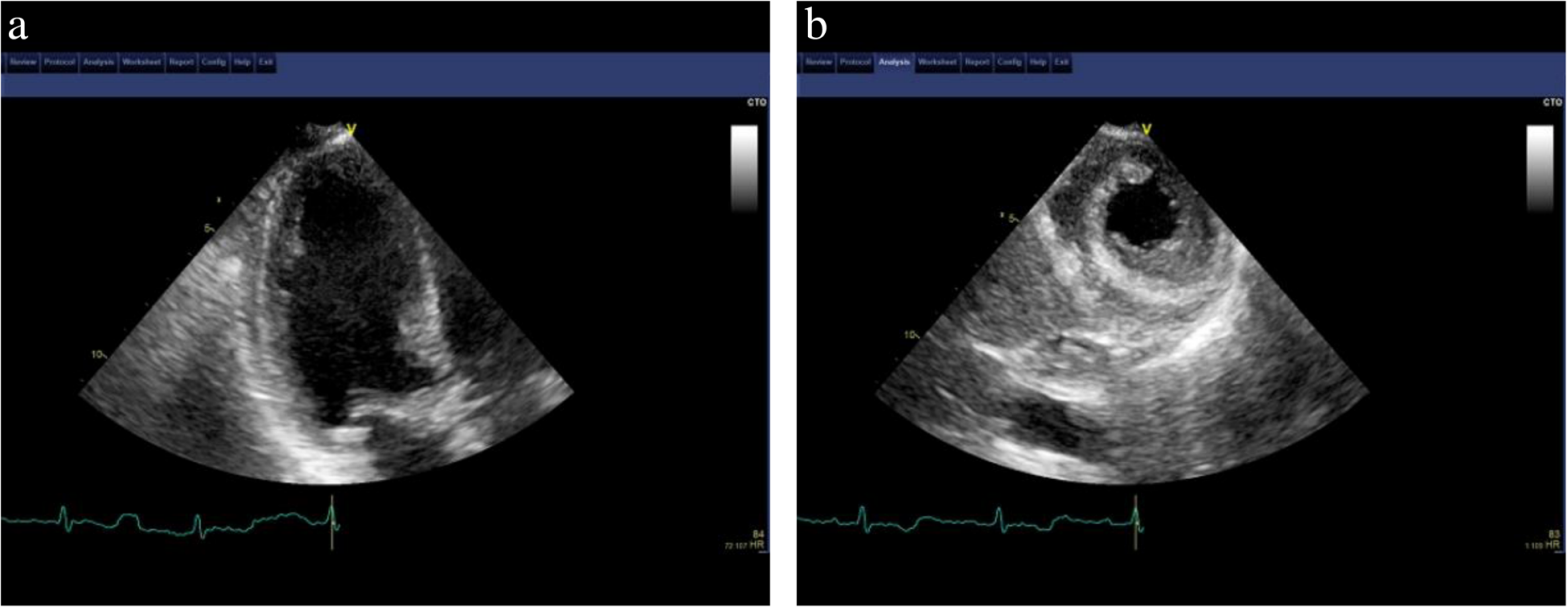
Transthoracic echocardiographic images. Long-axis (**a**) and short-axis (**b**) views obtained after 1 week of anticoagulant treatment showing complete resolution of the apical thrombus

**Fig. 4:**
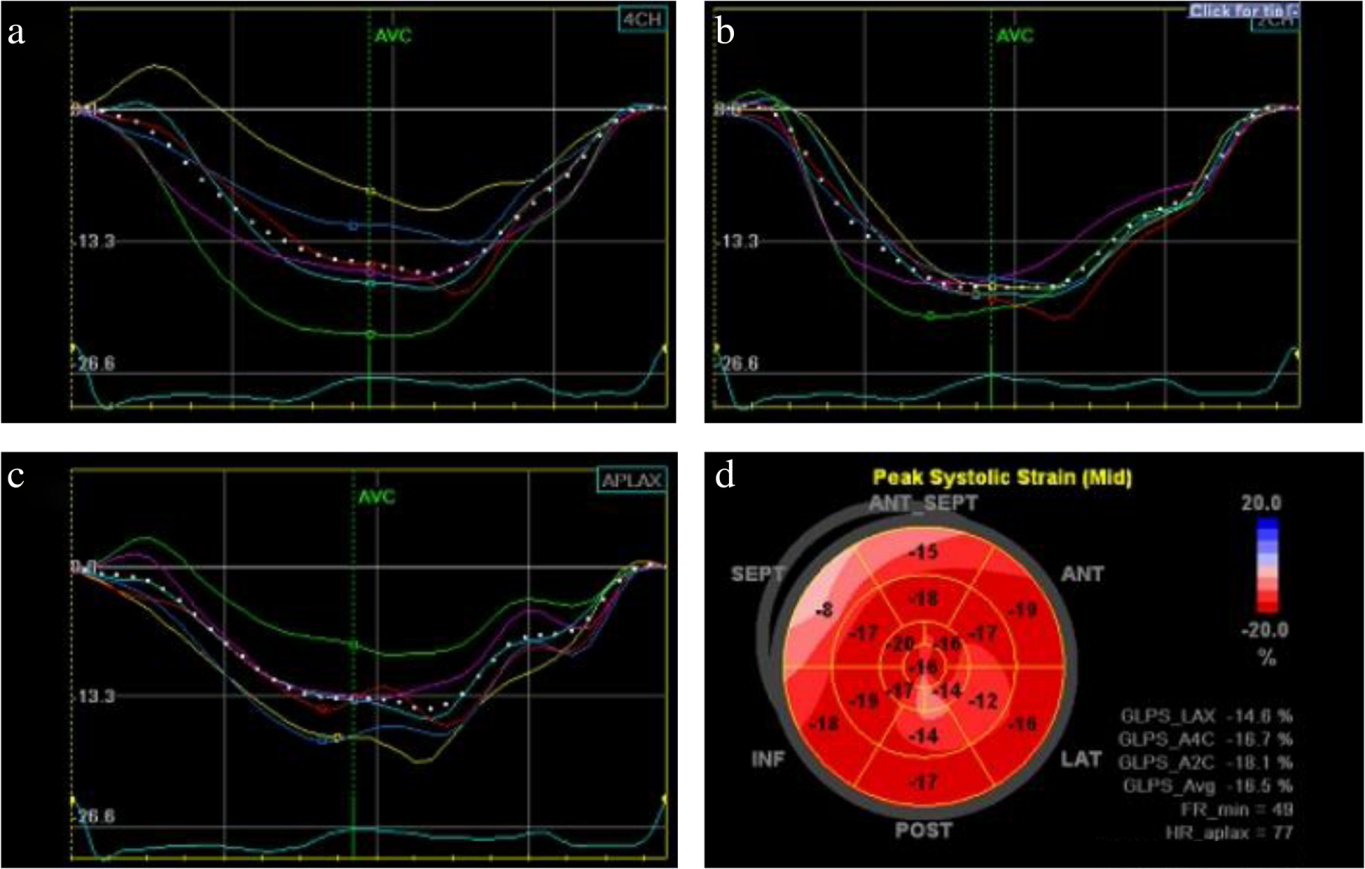
Longitudinal strain curves from four-chamber (**a**), two-chamber (**b**), and apical long-axis (**c**) views obtained by two-dimensional transthoracic echocardiography approximately 2 months after admission are used to generate bull’s-eye summary of the entire left ventricle (**d**). The bull’s-eye plot shows near normal left ventricular wall motion

On the seventh hospitalization day, although LV wall contraction slightly improved compared with the findings on admission, an approximately 10-mm thrombus was detected in the apex of the left ventricle by TTE (Fig. 2). Therefore, continuous intravenous administration of unfractionated heparin was initiated to maintain the activated partial thromboplastin time value 1.5–2.0-fold higher than the control value, and warfarin therapy was simultaneously initiated to maintain the prothrombin time international normalized ratio (INR) between 2 and 3. Anticoagulant therapy with warfarin was continued for 1 week until complete resolution of the apical thrombus (Fig. 3), and follow-up TTE confirmed that the systolic function of the left ventricle was completely recovered (Fig. 4). About 2 months after the admission, the patient was discharged to a retirement home for the elderly with a serum CK level of 47 U/L and a serum NT-proBNP level of 408 pg/ml. During the 7-month follow-up after discharge, no signs of TCM recurrence were observed, and the patient’s condition was stable.

### Patient 2

**Fig. 5:**
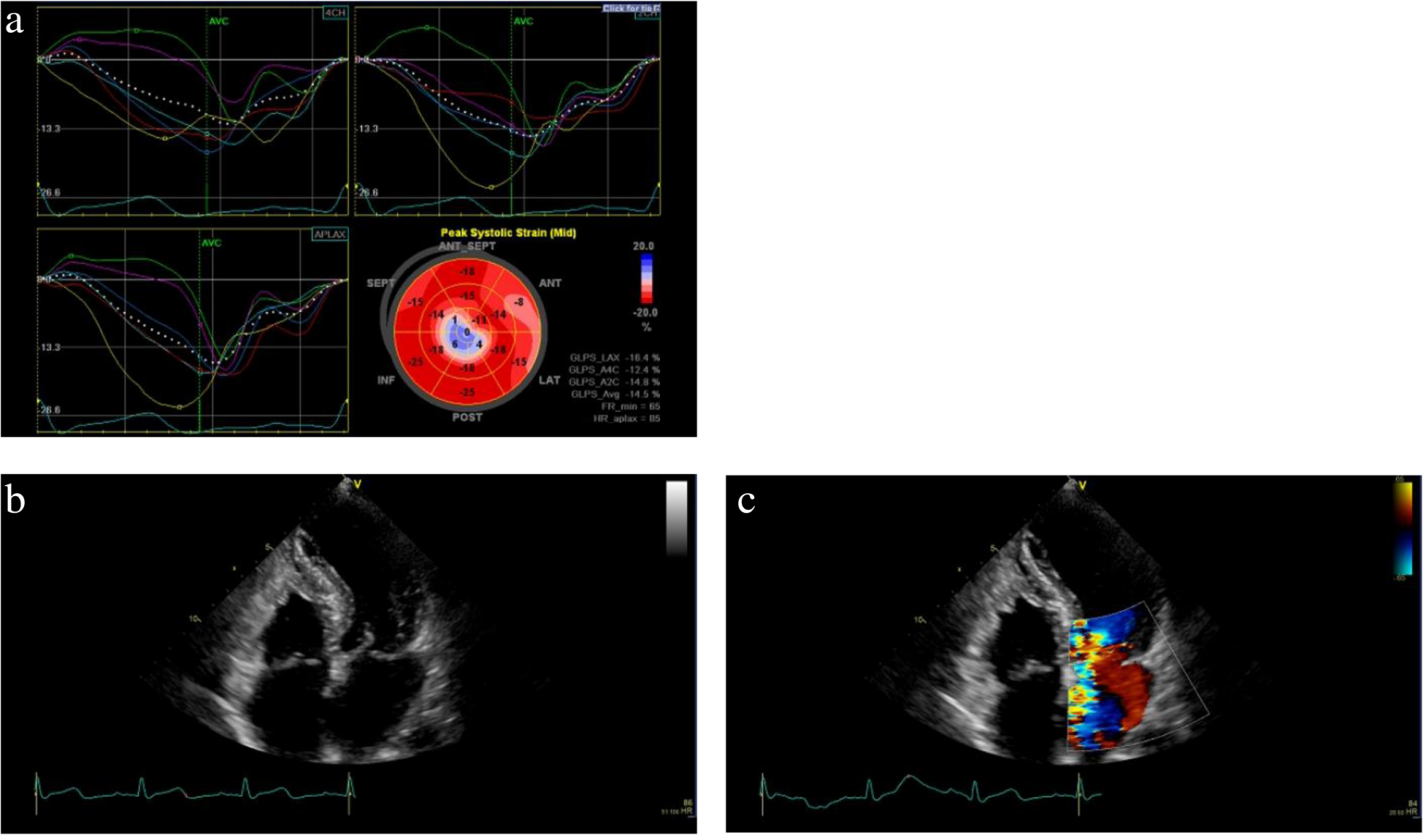
Transthoracic echocardiographic images obtained on admission showing (**a**) akinesis of the apical segments of the left ventricle, (**b**) tethering of the anterior mitral leaflet, and (**c**) eccentric severe mitral regurgitation

**Fig. 6:**
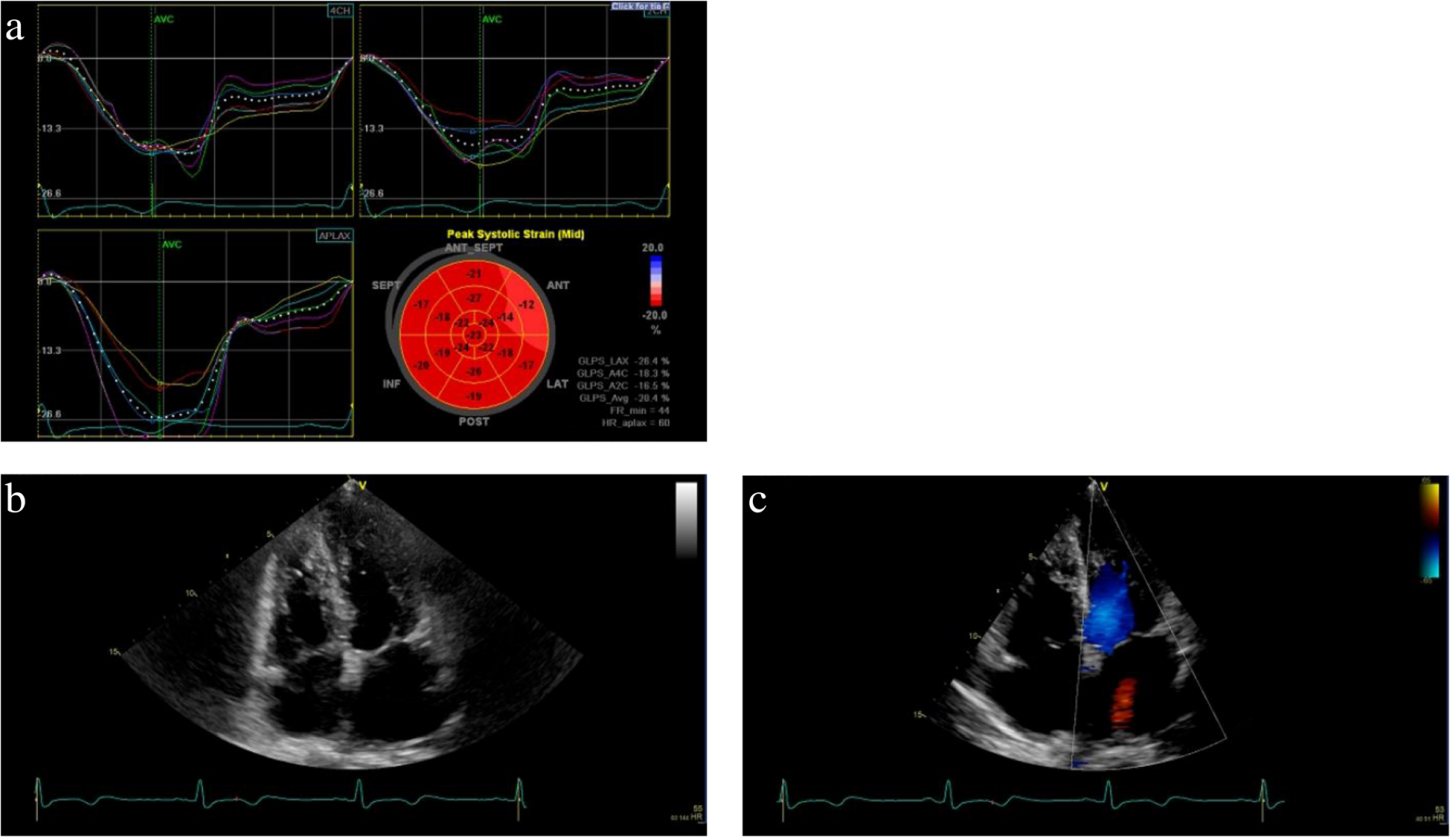
Transthoracic echocardiographic images obtained at 1 month after admission indicate (**a**) the recovery of left ventricular wall motion, (**b**) improvement of tethering, and (**c**) disappearance of mitral regurgitation

A 57-year-old Asian woman was referred to our hospital by her primary doctor because of persistent chest discomfort for 3 hours at rest. She had experienced similar symptoms intermittently in the previous 6 months. She had a medical history of hypertension and pneumonia. Her medication profile included valsartan 80 mg, amlodipine 5 mg, indapamide 0.5 mg, each taken once per day. She had no smoking habit, alcohol consumption, or family history of cardiac disease. She worked as a cook and transferred to her current workplace 1 year ago. She had experienced about 6 months of increasing work-related mental stress in a new managerial role. On physical examination, her pulse rate, blood pressure, and oxygen saturation were 88 beats/minute (regular), 119/84 mmHg, and 100%, respectively. Her body temperature was 36.2 °C. A grade 3/6 holosystolic murmur could be heard at the apical heart area as the loudest, whereas friction rubs and gallops were not heard. The rest of the examinations, including respiration and abdomen, were unremarkable. Edema was not detected in either lower limb. She was awake, alert, and oriented. Her neurological examination on admission did not reveal any motor or sensory deficit, and her cranial nerves were normal. ECG showed ST-segment elevation in V5 and V6 leads. The main laboratory findings were as follows: troponin T 1.04 μg/ml (normal range, < 0.1 μg/ml), CK 543 mg/dl (43–165 mg/dl), and NT-proBNP 2441 pg/ml (< 125 pg/ml). TTE showed apical LV wall akinesis with basal hyperkinesis and severe MR. She underwent an emergent cardiac catheterization. Coronary angiography showed no obstruction, and left ventriculography revealed an ejection fraction (EF) of 67.8%, severe localized apical hypokinesis with hyperkinesis of the basal segment, and grade 3 severe MR. Further two-dimensional echocardiographic examination with speckle tracking on the admission day demonstrated severe LV apical systolic dysfunction (EF, 59.7%) (Fig. 5a), severe MR caused by tethering of the anterior leaflet (Fig. 5b and c), and elevated pulmonary artery systolic pressure (38.5 mmHg), whereas neither left ventricular outflow tract (LVOT) obstruction nor systolic anterior motion (SAM) of the mitral valve was observed. The tenting length of the anterior mitral leaflet was 1.6 cm. On the basis of the aforementioned findings, the patient was diagnosed with TCM with a complication of severe MR. Because the hemodynamics of this patient were preserved, she could be managed with conservative observational treatment. The patient had an uneventful clinical course and was discharged in stable condition on the eighth day after admission. Follow-up TTE performed after 1 month revealed that tethering MR was almost resolved with complete resolution of LV wall motion abnormalities (Fig. 6), and laboratory data after 2 months were improved, with decreases in CK (154 mg/dl) and NT-proBNP (541 pg/ml). The patient had not experienced any apparent symptoms in 7 months of follow-up after discharge and had not been rehospitalized for the recurrence of TCM.

## Discussion

TCM is often described along with its cause, but there are few reports that mention its complications. We present two cases of transient complications in postmenopausal patients with TCM. Patient 1 was diagnosed with intraventricular thrombus, whereas patient 2 was found to have severe MR. TTE was very helpful in diagnosing these complications during the acute phase of TCM treatment. Patient 1 was started on oral anticoagulation with warfarin while bridging with unfractionated heparin until therapeutic INR was achieved and the disappearance of the thrombus was confirmed by follow-up TTE. On the other hand, patient 2 was managed with conservative treatment because the hemodynamic stability had been preserved. At approximately 1 month of follow-up, TTE showed reduction of MR with normalization of her LV systolic function.

TCM generally has an excellent prognosis; > 90% of patients recover completely, usually within 4–8 weeks but sometimes as long as 1 year [3]. However, TCM may be associated with potential complications, including cardiac rupture, LV thrombus, MR, various arrhythmias, and death. Based on a long-term follow-up study of patients with TCM, the rates of death of any cause and major adverse cardiac and cerebrovascular events were 5.6% and 9.9% per patient-year, respectively [4].

The incidence of cardiac thrombus due to TCM is reported to be 2.2–8.0% [5, 6]. In multivariate analysis including age, sex, LVEF, ST elevation at admission, and apical ballooning pattern, troponin I level > 10 ng/ml was the only predictor for the occurrence of LV thrombus [5]. LV apical thrombus formation may occur during the early stage due to transient apical asynergy combined with increased sympathetic activation, which accelerates the coagulation cascade [7, 8]. LV thrombus formation in TCM may be explained by the well-known Virchow triad that consists of the following three factors: blood stasis, endothelial injury, and hypercoagulability. Blood stasis is caused by hypokinesia of the middle and apical segments of the left ventricle with typical apical LV ballooning. Endothelial dysfunction, which could be explained by the propensity for microvascular coronary artery spasm, is common in patients with TCM, and it may be a pathogenetic mechanism for TCM. Attenuated endothelial function and increased catecholamine levels in patients with a prior episode of TCM were reported [1]. Cecchi *et al.* reported that patients with TCM had increased plasmin activator inhibitor 1 and von Willebrand factor levels, which lead to hypercoagulation [9]. Furthermore, a previous report showed a relationship between higher levels of epinephrine and elevated platelet activation or aggregation in patients with TCM [10]. These might be the reasons why patients with TCM develop apical thrombus in the acute phase. In our patient 1, the initial TTE showed only apical ballooning and akinesia without any evidence of LV apical thrombus; however, thrombus formation occurred after 1 week. Although a severe complication due to LV thrombus is known to be systemic embolization, such as in cerebral, renal, and peripheral limb arteries, any embolic events in this type of patient may be prevented by early anticoagulant therapy. Otani *et al.* reported that the frequency of cerebral infarction due to TCM ranged from 0% to 9.5%, and it was similar or higher than the frequency of stroke after atrial fibrillation (4.0–9.0%) or myocardial infarction (4.6%) [11]. A report by Gregorio showed that cerebrovascular thromboembolic events occurred in 33% (25% presented as stroke) of patients with TCM with LV apical thrombus [12]. The thrombus in the left ventricle has been classified as mural or protruding, with prevalence of 40% or 60%, respectively [13]. A mural thrombus is flat and parallel to the endocardial surface of the myocardium. A protruding thrombus, which is usually spherical and mobile, is thought to be associated with an increased risk of ischemic stroke. Patients with definite thrombus or those with large akinetic segments of the left ventricle should be considered for anticoagulant treatment. However, there is no definitive clinical guideline regarding anticoagulant therapy for the management of intracardiac thrombus in TCM. A European position paper recommends oral anticoagulation when intraventricular thrombus is detected in high-risk patients with TCM in the absence of high bleeding risk [14]. Although the optimal duration of anticoagulant therapy for that complication also remains under debate, apical thrombus resolution and LV function recovery should be documented before anticoagulation is withdrawn. In this case, we had continued anticoagulant therapy for patient 1 until full resolution of the patient’s abnormal LV wall motion. In addition, surgical management may also be considered because there is an increased risk of embolism if thrombus remains after anticoagulation therapy, even with improved wall motion. Suzuki *et al.* reported a case of a patient with TCM-related LV protruding thrombus requiring surgery [15].

Significant (moderate-to-severe or severe) acute MR is another potentially serious complication, accounting for 8–19% of patients with TCM [16, 17]. In multivariate analysis, LVEF on admission and mitral SAM were the only predictors of acute MR in patients with TCM [17]. Patients with significant MR have lower LVEF and higher pulmonary artery pressure, which may lead to acute heart failure and cardiogenic shock. Therefore, early detection by using TTE is important to providing appropriate management. Significant MR is commonly reported in patients presenting with apical ballooning, which is a representative form of TCM. MR can be observed with or without mitral SAM. The reason underlying the occurrence of MR in patients with TCM has not been completely elucidated, but the following two distinct underlying mechanisms concerning significant acute MR associated with TCM are considered: (1) the coexistence of SAM and LVOT obstruction and (2) tethering of the mitral valve leaflets [18]. Dynamic SAM and LVOT obstruction may occur in the acute stage of TCM, with a prevalence of up to 33% reported previously [19]. SAM-induced LVOT obstruction involves multiple complex factors, such as apical ballooning, distortion of the left ventricle, mitral valve displacement, septal bulging, and hypercontractile basal LV segments [17]. Because LVOT obstruction and severe MR increase the risk of cardiogenic shock and pulmonary edema, they must be properly and quickly diagnosed to ensure optimal clinical outcomes. In this case, TTE revealed neither LVOT obstruction nor SAM. In contrast, a significant tethering of the anterior mitral leaflet, which was considered the cause of the eccentric severe MR jet, was confirmed by transesophageal echocardiography. Because MR was almost resolved, based on the improvement in the LV apical contraction, tethering of the anterior mitral leaflet was thought to be due to apical ballooning of TCM in patient 2. The main factor associated with severe MR without SAM may be the displacement of the papillary muscle, which leads to impaired leaflet coaptation secondary to tethering [20]. Quantitative evaluation of mitral valve morphology, including the papillary muscle, requires multidetector computed tomography (MDCT) or real-time three-dimensional echocardiography, but these modalities were not used in this case. A previous study indicated that MDCT has been valuable in evaluating precise mitral valvular and LV geometry, including papillary muscle positions [21]. The distance between posterior papillary muscle tips and anterior annulus has been reported to be a major determinant of functional MR severity [22]. Generally, severe mitral valve tethering is considered an important cause of ischemic MR. Similar to ischemic MR, tethering by papillary muscle displacement due to LV dysfunction has been considered another mechanism of functional MR in patients with TCM. An echocardiographic study evaluating the mechanism of acute MR in 47 patients with TCM reported that 12 of 47 (25.5%) had moderate-to-severe MR and 6 of 12 (50%) had SAM and LVOT obstruction, whereas the remaining 6 of 12 patients without SAM showed tethering of the mitral valve [18].

There are no randomized clinical trials to establish specific treatment of TCM. In patients with mild TCM, no treatment or a short course of limited medical therapy may be sufficient. Renin-angiotensin-aldosterone system blockers may be considered during the period when regional wall motion abnormality is present in patients with heart failure with reduced EF. Administration of β-blockers would be reasonable when coronary spasm is not suspected as the etiology of TCM, because catecholamine is often excessive. When the patients with TCM have severe congestive heart failure or cardiogenic shock, it is important to assess whether significant MR or LVOT obstruction is associated with their condition. Cardiogenic shock, which occurs in 4–20% of patients with TCM, may be exacerbated by right ventricular involvement, LVOT obstruction, or acute MR [14]. In patients with hemodynamically significant LVOT obstruction, the use of β-blockers should improve the gradient of LVOT obstruction by reducing basal hypercontractility and heart rate and prolonging the diastolic LV filling period. In more severe cases with progressive end-organ dysfunction because of cardiogenic shock, early mechanical support should be considered as a bridge until the recovery of cardiac function.

## Conclusions

Intracardiac thrombus and severe MR may be causes of embolic events and cardiogenic shock, respectively, which may lead to poor clinical outcomes. Therefore, detection and diagnosis of the presence of complications, especially in the acute phase, and appropriate treatment of the complications are necessary for patients with TCM.

## Abbreviations

CK: Creatine kinase
ECG: Electrocardiogram
EF: Ejection fraction
LV: Left ventricular
LVOT: Left ventricular outflow tract
MDCT: Multidetector computed tomography
MR: Mitral valve regurgitation
NT-proBNP: N-terminal pro-B-type natriuretic peptide
PT-INR: Prothrombin time international normalized ratio
SAM: Systolic anterior motion
TCM: Takotsubo cardiomyopathy
TTE: Transthoracic echocardiography
